# Haemorrhaging lesion in the breast: is there a role for embolisation?

**DOI:** 10.2349/biij.2.3.e30

**Published:** 2006-07-01

**Authors:** NA Taib, CH Yip, S Ranganathan, F Moosa, KS Mun

**Affiliations:** 1 Department of Surgery, Faculty of Medicine, University of Malaya, Kuala Lumpur, Malaysia; 2 Department of Radiology, Faculty of Medicine, University of Malaya, Kuala Lumpur, Malaysia; 3 Department of Pathology, Faculty of Medicine, University of Malaya, Kuala Lumpur, Malaysia

## Abstract

Angiosarcoma of the breast is an extremely rare condition. This case illustrates the use of embolisation as a modality of treatment for primary breast angiosarcoma. No other case has been reported on the use of embolisation for this disorder.

## INTRODUCTION

Angiosarcoma is the most malignant amongst all vascular tumours. This entity is also known as haemangiosarcoma, haemangioblastoma, and metastasizing haemangioma. Diagnosis of this lesion usually translates to a lethal disease. Common sites that have been reported are skin, soft tissue, breast, liver, and spleen. Angiosarcoma of the breast is a very rare condition. At our centre, this was the only case seen over a period of 10 years. Compared with primary angiosarcoma, secondary angiosarcoma from a previously irradiated breast is more commonly reported.

## CASE REPORT

**Figure 1:**
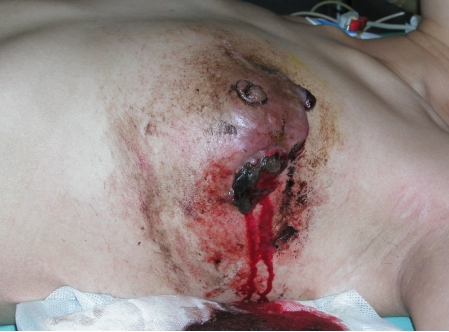
Haemorrhaging lesion of the breast.

**Figure 2:**
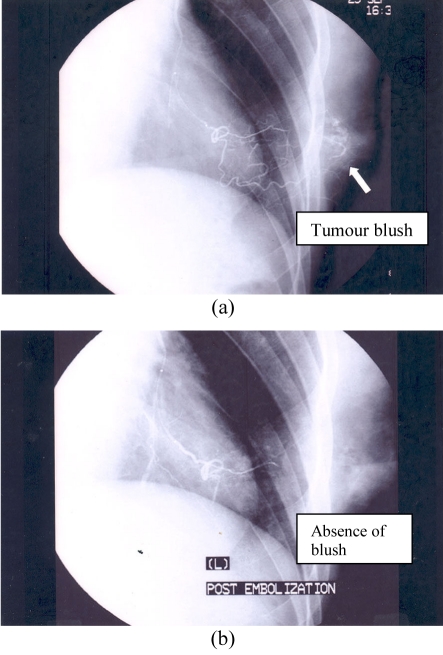
(a) Pre-embolisation angiogram of the left breast; (b) Post-embolisation angiogram of the left breast.

The patient was a thirty-year old clerk. She complained of an abnormal warm sensation in her left breast. She consulted her doctors regarding this problem, but was reassured that it was normal. Sixteen months later she noted a definite lump in her left breast, and an excision biopsy was done. The lump recurred two months later, and a similar procedure was performed. Both biopsies were reported as benign haemangiomas. The lesion recurred during the next two months and, this time, she approached our clinic. Physical examination revealed a mass which measured approximately 6 cm. She was advised mastectomy with immediate breast reconstruction. She refused, but one month later, returned to our hospital with a haemorrhaging lesion in the left breast (Figure 1). She was found to be pale, but was haemodynamically stable. She underwent radiological embolisation of the lesion. The angiogram showed a tumour blush that had feeding arteries from branches of the left internal mammary artery and lateral thoracic artery. Embolisation of the feeding vessels was done using polyvinyl alcohol (PVA). Post-embolisation images showed complete occlusion of these feeding vessels (Figures 2a, 2b). She later consented for mastectomy. The tumour was completely excised and was reported as a grade 2 angiosarcoma. Radiotherapy of the chest wall was given, and investigations showed multiple lung metastases and bone metastases. She, however, refused palliative chemotherapy. Five months after mastectomy, she returned with right hypochondrium pain and abdominal distension. On clinical examination, she was pale. Her haemoglobin was 6 g/dl with a platelet count of 83 x 10^9^/L. The low platelet count could be attributed to continuing platelet consumption due to formation of thrombi in the vascular metastatic lesions. Her abdomen was found to be grossly distended, tense, and tender. The fluid thrill test was positive. CT scan of the abdomen revealed multiple liver metastases with free fluid in the peritoneal cavity. The main hepatic artery was successfully embolised. Palliation of symptoms was achieved with analgesics, repeated peritoneal tapping, and blood transfusions. The patient also developed minor local recurrence on the chest wall, which fortunately did not bleed. Seven months after treatment and 28 months after initial excision biopsy, she deteriorated rapidly and passed away.

## DISCUSSION

The peak incidence of angiosarcoma of the breast is usually in the fourth decade of life. The overall survival is poor; five–year survival is only 33% [1]. Many studies show that prognosis is closely correlated with the histological grade of the tumour [2]. Misdiagnosis appears to be a common problem with angiosarcoma [3], illustrated perfectly by this case. Histological misdiagnosis has been reported to be as high as 39% in cases involving the first biopsy [1]. Angiosarcoma must be ruled out in all vascular lesions of the breast. Angiosarcoma can be divided into three histological grades [2]. The tumour may exhibit features of more than one histological grade, i.e., one area may show a lower grade or even benign features, while other areas in the tumour may show malignant features. Adequate sampling is essential to ensure accurate diagnosis. In this case, previous histopathological slides were not available for review. It may have been that the areas sampled in the two lesions excised first showed a grade 1 tumour, which could be easily mistaken for a benign lesion. Clinical presentation of angiosarcoma may range from a lump to an overtly bleeding lesion. It may also present as a painless mass or general enlargement of the breast without a definite mass. Many cases show involvement of both breasts [1]. Our patient reported a “warm” sensation in the breast before any lump could be detected. In this case, diagnosis of angiosarcoma at an earlier stage may not have altered the unfortunate end result as she was not compliant to treatment. The radiographic features of breast angiosarcoma are not specific. Mammogram was not done for this patient as the mass bled easily even with the lightest compression. To arrest the bleeding, embolisation was performed, but this was only a temporary measure. Eventually she agreed to mastectomy, which was the definitive treatment. No other case has been reported so far on a Medline search for embolisation of angiosarcoma of the breast as a form of treatment for primary angiosarcoma of the breast. There are, however, three reports of embolisation treatment for primary hepatic angiosarcoma [4-6]. Invasive procedures are usually not carried out in patients with metastatic disease, but in this patient, embolisation of the main hepatic artery was indicated as she continued to bleed intraperitoneally. The role of adjuvant therapy (chemotherapy and/or radiotherapy) for breast angiosarcoma is still unclear, due to the small number of cases seen [3].

## CONCLUSION

Angiosarcoma is a rare, but highly aggressive tumour. As shown by this case, all vascular lesions in the breast warrant a careful workup to rule out angiosarcoma. Embolisation may have a limited role in the management of primary breast angiosarcoma and metastases in the liver. Therefore, a multidisciplinary approach is crucial to the management of this condition.
